# Effects of cod intake in pregnancy on iodine nutrition and infant development: study protocol for Mommy’s Food - a randomized controlled trial

**DOI:** 10.1186/s40795-018-0215-1

**Published:** 2018-02-17

**Authors:** Maria Wik Markhus, Ingrid Kvestad, Lisa Kolden Midtbø, Ive Nerhus, Elisabeth R. Ødegaard, Ingvild Eide Graff, Øyvind Lie, Lisbeth Dahl, Mari Hysing, Marian Kjellevold

**Affiliations:** 1 Institute Institute of Marine Research, PO Box 1870, Nordnes, 5817 Bergen, NO Norway; 2 Regional Centre for Child and Youth Mental Health, Uni Research Health, Uni Research, PO Box 7810, 5020 Bergen, Norway; 3 Uni Research Health, Uni Research, Bergen, Norway

**Keywords:** Iodine intake, Iodine status, UIC, Maternal diet, Nutritional status, Fish, Neurodevelopment, Bayley scales of infant and toddler development, RCT, Pregnancy, Infant nutrition

## Abstract

**Background:**

Iodine is a key component of thyroid hormones that are critical for normal development of the brain and nervous system in utero. Recent results indicate that two thirds of pregnant women in Europe have sub-optimal iodine nutrition. In Norway, milk and seafood are the most important dietary iodine sources and contributes to about 80% of the intake.

**Method:**

Two-armed randomized trial where 137 pregnant women were randomized to either receiving cod twice weekly, or continue with habitual diet for 16 weeks (pregnancy week 20–36). Socioeconomic- and demographic factors, dietary information and biological (urine, blood, and hair) samples are collected pre- and post-intervention, and at six weeks, three-, six-, and eleven months postpartum. Biological samples (urine, blood, and hair) of the infant are collected at six weeks, three-, six-, and eleven months postnatal. Child development is assessed by The Bayley Scale of Infant and Toddler Development, 3rd edition, at eleven months, and by parent report on the Ages and Stages Questionnaire, 3rd edition, and Ages and Stages Questionnaire: Social Emotional at three-, six-, and eleven months.

**Discussion:**

The Mommy’s Food study will provide knowledge on changes in iodine nutrition when consuming iodine rich fish during pregnancy and contribute to the understanding of the impact of iodine status in pregnancy on infant neurodevelopment.

**Trial registration:**

This study is registered in ClinicalTrials.gov (NCT02610959). Registered November 17, 2015.

## Background

Iodine is an essential nutrient for the synthesis of thyroid hormones triiodothyronine (T_3_) and thyroxine (T_4_). Dietary iodine is rapidly absorbed through the stomach and duodenum, and the thyroid gland is dependent on regular and adequate supply through the diet in order to produce these vital thyroid hormones. Thyroid hormones are involved in a range of biochemical processes, including regulation of metabolic rate, energy production, protein and enzyme synthesis, thermoregulation, growth and neural development [1].

The iodine requirement during pregnancy is greatly increased because the mother synthesizes approximately 50% more T_4_ to maintain maternal euthyroidism and to transfer thyroid hormones to the fetus prior to gestational week 20, and because iodine needs to be transferred to the fetus for fetal thyroid hormone production post gestational week 20. In addition, the renal iodine clearance increases during pregnancy. Thus, it is important for pregnant women to consume sufficient iodine during pregnancy [1].

In general, we have limited dietary iodine sources and the iodine content of terrestrial foodstuffs is much lower compared to marine foodstuffs. The only good natural source of iodine is seawater fish and other marine products [2]. Iodized salt is the most important source worldwide and is also the agreed strategy for achieving iodine sufficiency [2]. In Norway, iodized salt contributes insignificantly as the maximum level of iodine added to salt is 5 μg/g, and the food industry is not permitted to use iodized salt.

Pregnant and lactating women are vulnerable groups in terms of developing iodine deficiency since they have higher needs of iodine [2, 3]. This has been confirmed in the most recent data from the Norwegian Mother and Child Cohort Study (MoBa) (*n* = 61,904), demonstrating inadequate iodine intake in the majority of pregnant Norwegian women. The median iodine intake was 141 μg/day from food and 166 μg/day from food and supplements. Only 21.7% reach the recommended daily intake by WHO/UNICEF/ICCIDD of 250 μg iodine/day, and the study concludes that suboptimal iodine intake among Norwegian pregnant women is a health concern [4]. Determination of iodine status by urinary iodine concentration (UIC) in a sub-study of the MoBa (*n* = 119) was in line with the findings from the dietary study [4], and insufficient iodine status has also been demonstrated in other studies among Norwegian pregnant women [5, 6].

Inadequate iodine status in pregnancy has been associated with suboptimal cognitive outcomes in the children at eight years of age in observational studies [7]. This in line with the MoBa study where maternal iodine intakes below the Estimated Average Requirements (160 μg/d) during pregnancy was associated with child language delay, impaired fine motor skills and mental health problems at three years of age, but no associations with gross motor skills as measured [8]. Interestingly, the results showed no evidence of a protective effect of iodine supplementation during pregnancy on child development. Few intervention studies on maternal iodine supplementation on child development exist and the quality of the existing studies are poor, as concluded in a recent review [9]. To our knowledge, this is the first trial intervening pregnant women with iodine rich foods (cod), to study its effects on maternal iodine status and infant development.

## Methods/design

The overall aims of this trial are to investigate, in a randomized controlled trial (RCT), if an increased intake of cod in pregnancy has an impact on (1) maternal iodine status and (2) infant development.

### Trial design

The study is a two-armed randomized controlled intervention trial involving cod intake in pregnancy with the primary purpose of prevention. The ideal control would be pregnant women from the same trial whom were to be instructed to continue with their habitual diet with restriction to include iodine. For ethical reasons the control is pregnant women, within the same trial, who will continue with their habitual diet. The primary outcome is UIC measured at post intervention (gestational week 36), and the secondary outcome is neurodevelopment assessed by the cognitive, language and motor scales of the Bayley Scales of Infant and Toddler Developmental 3rd edition (Bayley-III) when the infants are 11 months of age. Other outcomes include urine samples, dietary data and blood samples from the mother and from the infant, as well as data on child development such as developmental status, socio-emotional abilities and infant sleep, and maternal mental health, that will be collected at several time points during the first year of life.

### Study setting and recruitment

**Fig. 1:**
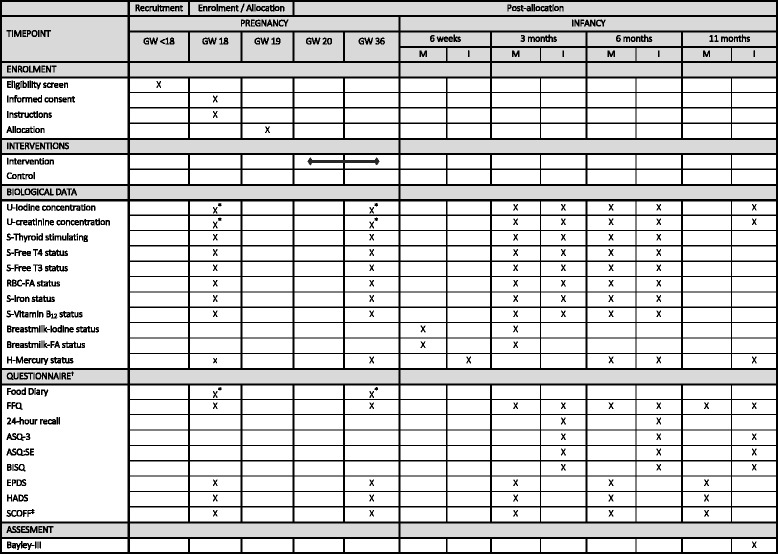
Overview of study schedule and main activities. *Six consecutive days between GW 18 and GW 19 and between GW 35 and GW 36 (last days of intervention meal). †Questionnaire includes: participant characteristics; self-reported maternal mental health; and parent-reported infant mental health. Abbreviations: GW, gestational week; M, mother; I, infant; U, urine; S, serum; T4, thyroxine; T3, triiodothyronine; RBC, red blood cells; FA, fatty acids; H, hair; FFQ, Food Frequency Questionnaire; ASQ-3, The Ages and Stages Questionnaire; ASQ:SE, The Ages and Stages Questionnaire: Social Emotional; BISQ, Brief Infant Sleep Questionnaire; EPDS, The Edinburgh Postnatal Depression Scale; HADS, The Hospital Anxiety and Depression Scale. ^‡^Screening tool for eating disorders

Participants were recruited through the Women’s clinic at Haukeland University Hospital in Health region west in Norway. Approximately 5000 women give birth at the Women’s Clinic every year, which is the local hospital for the population in Bergen and surrounding areas. From January 2016 until February 2017, information regarding the intervention trial was included in the postal mail, sent from the Women’s Clinic, with the date/time for the routine ultrasound (RU). In Norway, the RU takes place in gestational week 17–19. To ensure sufficient participants information regarding the trial and invitation to participate was also broadcasted online (Facebook, Instagram, and magazine for pregnant in Norway). Pregnant women who were interested in the study contacted the secretariat. Time schedule of enrolment, interventions, assessments, and all visits for participants is schematically diagrammed in Fig. 1.

### Eligibility criteria

Inclusion criteria: prim parous singleton pregnancy ≤ gestational week 19, Norwegian speaking and/or understand Norwegian writing (all validated tests of the child will be in Norwegian). Exclusion criteria: allergies to fish, chronic diseases known to affect iodine status (Graves’ disease, Thyroiditis, Thyroid Nodules, known hypothyroidism or hyperthyroidism).

### Randomization, allocation and blinding

Randomization will be performed individually by lottery at the second visit in gestational week 19 in blocks of 10 to ensure approximately equal allocation to the two study groups. Study investigators will enroll participants, generate the allocation, and randomly assign participants to interventions after baseline sampling. The study id-number for each participant will be a random number between 1 and 200. By this procedure, participants and investigators will be blinded to the allocation until the baseline sampling has been completed. Owing to the nature of the intervention, blinding of the participating mothers will not be possible. However, the infants will be blinded throughout the trial and data analysis and study investigators and laboratory personnel will be blinded when analyzing data.

### Dietary intervention

#### Study foods and instructions

Participants in the intervention group will receive frozen cod fillets (Lerøy A/S, Bergen, Norway) and are asked to consume two intervention meals of 200 g weekly (a total of 400 g cod per week). They will prepare the dinner meal themselves and are free to choose recipes. However, they will be provided with recipes that could be used ad libitum. For compliance purposes, participants will be instructed to weigh (Kitchen Scale, article no. 34–1207-16, ClasOhlson.com) the cod fillet before preparing the meal, and weigh the fillet leftovers (if any), after the meal is completed. The participants will record these data, in addition to receipt used and date of consumption, in a weight registration form. These data will be used in order to calculate iodine intake from the intervention meal. The participants in the control group will be instructed to follow their habitual diet. Once a pregnant woman is enrolled or randomized, the study investigators will make every reasonable effort to follow the participant for the entire study period. Prior to all the visits, the participants will be reminded about the upcoming appointment. Study investigators will always take into account the participants’ private calendar in the attempt to best ensure retention.

#### Safety of foods

Women in the intervention group will have an intake of (0.4 kg cod per week * 0.04 mg Hg/kg *1000=) 16 μg mercury (Hg) per week from the study diet. Weekly intake of dioxin and dioxin-like polychlorinated biphenyls (dl-PCBs) will be (0.4 kg per week *0.08 ng toxic equivalences (TEQ) /kg* 1000=) 32 pg TEQ per week. The CONTAM panel in the European Food Safety Authority (EFSA) established a tolerable weekly intake (TWI) for organic Hg of 1.3 μg/kg bodyweight. This is in accordance with the Joint FAO/WHO expert Committee on Food additives (JECFA) [10]. The equivalent TWI for dioxin and dl-PCB is 14 pg TEQ per kilo bodyweight [11]. For the participants in the study, their TWI is calculated based on the 5-percentile weight of the women in the Little in Norway cohort, *n* = 828 (unpublished data) (5 percentile = 56 kg). That means that they have a TWI of 71.2 μg Hg per week and 784 pg TEQ for dioxin and dl-PCBs. Thus, the intake of Hg and dioxins and dl-PCBs will be maximum 22% and 4% of the TWI. Consequently, the intake of cod in this study is far below the TWI for this especially vulnerable population.

### Assessment and data collection

#### Biological samples-urine

Participants will receive six marked tubes for collection of urine in gestational week 18–19 and in week 35–36 together with instructions on how to collect the spot urine samples. A pooled sample of 1 ml of homogenized spot urine collected on six consecutive days, between 4 pm and midnight, will be analyzed for iodine pre- and post intervention in both groups. The participants will keep the urine samples in their home freezer until the visits in gestational week 19 and 36. At this visits at three and six months postpartum, the mothers will deliver a spot urine sample. For the infants, urine collection pads will be placed in the diapers 1–2 h before the visit for urine sampling, and extracted at the visit at three-, six and eleven months. The urine samples will be transferred to cryo tubes and stored at minus 20 °C pending analysis by inductively coupled plasma mass spectrometry (ICP-MS). Method description of UIC (μg/L) determination is previously described by Dahl et al. [12]. Urinary creatinine concentration (g/L) will be analyzed using MAXMAT PL II multidisciplinary diagnostic platform with a creatinine PAP kit [13].

#### Biological samples-blood

Pre- and post-intervention blood sampling (gestational week 18 and 36) will include thyroid hormone status (thyroid stimulating hormone (TSH), free T_3_, free T_4_), fatty acid status, iron status and vitamin B_12_ status. The same blood sampling will be conducted in mothers and infants at three- and six months postpartum. Venous blood for serum preparation will be collected in BD Vacutainer® SST™ vials II *Advanced* and set to coagulate for minimum 30 min before centrifuging (1000–1300 G, 20 °C, 10 min) within 60 min. Venous blood for plasma preparation will be collected in BD Vacutainer® K2E 5.4 mg (adults) and 3.6 mg (infants) vials and centrifuged (1000–1300 G, 20 °C, 10 min) within 30 min, for the preparation of erythrocytes. Erythrocytes will be adequately separated from plasma and the layer on top of the cells (buffy coat) to ensure a clean erythrocyte fraction. In cases were venipuncture of infants will be problematic, capillary blood will be collected from the heel or a fingertip, depending on age/weight. Prior to capillary blood sampling, the heel or finger will be warmed with a hot water balloon to ensure vasodilation to enable sufficient blood flow. Tenderfoot ITC® heel incision devise will be used for heel pricks and ACCU-CHEK® Safe-T-Pro Plus lancet will be used for finger pricks. Blood samples will be stored at minus 80 °C, pending analysis. TSH, free T3, and, free T4 will be analyzed in serum using magnetic separation and detected by chemiluminescence labeled with acridinum ester on an Advia Centaur XPT (Siemens Healthcare diagnostics Inc., Tarrytown, USA). Ferritin will be analysed by an immunoturbidimetric method using Advia Chemistry XPT (Siemens Mediacal Solutions Diagnostica, Japan). Fatty acids will be analyzed in red blood cells by standardized procedures at Institute of Marine Research (IMR) [14], using ultrafast gas chromatographic (UFGC) (Thermo Electron Corporation, Massachusetts, USA). In case of advances concerning biomarkers of the primary or secondary outcome variable, other analysis than those already mentioned will be considered. This includes single nucleotide polymorphisms (SNPs) analyses from buffy coat.

### Biological samples-breastmilk

At six weeks and three months postpartum, the mothers are requested to sample breastmilk or formula. The breastmilk or formula will be analyzed for iodine content and fatty acids. Participants will receive instructions and six marked cryo tubes for collection of breastmilk at the start, during and the end of a chosen feed for two days. If the mother is not breastfeeding, she will prepare a meal of the given formula and collect in two tubes for two days. Mothers will be instructed to record date and time of sampling, and store the samples in their home freezer pending pick-up from study investigators (samples from six weeks postpartum) or submission of samples at the three months follow up visit. For submission of samples, the participants will receive freezer packs for transportation. The samples will be stored at minus 80 °C pending analysis for iodine concentration on ICP-MS, previously described by Dahl et al. [15], and fatty acids by standardized procedures at IMR [14], using ultrafast gas chromatographic (UFGC) (Thermo Electron Corporation, Massachusetts, USA).

### Biological samples-hair

Hair samples will be collected for mercury analysis from the mothers in gestational week 18 and 36, and at six months postpartum, and from infants at age six weeks, six- and eleven months. Mothers following instructions at age six weeks, will perform hair collection from infants. Samples are obtained from the mothers and infants by cutting a hair-bundle with an approximate diameter of 2–5 mm, as close to the scalp as possible from the occipital area of the head. A thin thread will be tied closest to the end nearest the scalp. Hair samples will be stored in room temperature pending analysis using Direct Mercury Analyser (DMA-80, Milestone) [16].

### Dietary intake

A structured 6-days food diary (FD), developed for this study with the purpose to estimate maternal iodine intake, will be handed out at the first visit, in gestational week 18, and mailed by post to be filled out in gestational week 35. The habitual diet of both intervention and control group is reported in a short Food Frequency Questionnaire (FFQ) in gestational week 18 and 35, and three-, six, and eleven months postpartum. The FFQ captures iodine rich foodstuff (seafood, milk, dairy, eggs and supplements), is based on a validated short seafood FFQ developed to analyze food habits in pregnant and postpartum women [17].

Infant’s dietary intake will be collected through non-validated FFQ’s (custom to the infants’ age) at three-, six- and eleven months of age. In addition, a 24-h diet recall interview will be conducted at the three- and six months’ visit. The mothers will be asked what the infant has ingested during the last 24 h. In cases of non-report of supplement intake, the mothers will actively be asked if the infant has been given any dietary supplements.

### Infant development

At eleven months, the children will be assessed with the Bayley-III by trained study investigators. The Bayley-III is a comprehensive assessment tool of neurodevelopment administered directly with the child. The tool includes three main subscales; the cognitive, language (receptive and expressive) and motor (fine and gross motor) scale. The Bayley-III represents the gold standard of developmental assessments in this age group and is widely used as an outcome measure in clinical trials. Two testers will administer the Bayley-III after undergoing training by a clinical child psychologist and a neuropsychologist. Standardization exercises will be conducted measuring the inter-rater reliability of the assessor compared with a gold standard until satisfactory level of agreement. During the trial, 10% of the tests will be double scored, and inter-rater agreement will be assessed to ensure high quality testing.

In addition, child development will be measured at three-, six- and eleven months by The Ages and Stages questionnaire, 3rd edition (ASQ-3) [18]. The ASQ-3 is an age-specific parent-reported screening questionnaire consisting of 30 items covering five developmental domains and has five subscales: communication, fine-motor, gross-motor, problem-solving, and personal-social. The scale has been translated and validated to a Norwegian setting [19].

### Infant mental health and sleep

Measures in infant socio-emotional abilities and sleep is included in the electronic questionnaires at three- six-, and eleven months postpartum. Socio-emotional abilities will be measured by The Ages and Stages Questionnaire: Social Emotional (ASQ:SE) [20]. The ASQ:SE is a short parent-reported questionnaire on social and emotional development in children, which has demonstrated good psychometric properties in the United States [21] and Norway [19]. Infant sleep is measured by the Brief Infant Sleep Questionnaire (BISQ). BISQ is a short questionnaire on infant sleep that has demonstrated good psychometric properties as a sleep screening tool for clinical and research purposes in infants and toddlers [22].

### Maternal self-reported mental health.

Data regarding maternal self-reported mental health is included in the electronic questionnaires pre- and post-intervention (gestational week 18 and 36, respectively), three- six-, and eleven months postpartum. Mothers will be asked questions regarding sleep duration and insomnia symptoms. The Relationship Satisfaction Scale [23] will be used to measure relationship satisfaction. The Edinburgh Postnatal Depression Scale (EPDS) [24] is included as a screening for postnatal depression, and the Hospital Anxiety and Depression Scale (HADS) [25] is included as a screening for anxiety and depression. And finally, the SCOFF questionnaire will be used to screen for eating disorders among the mothers [26].

### Data management and biobank

All collected data will at all times be treated strictly confidential and data will only be used pseudonymized for all evaluations. The protocols and questionnaires from the cognitive assessments will be administered at Uni Research Health. Scoring of the protocols will be done on a secure computer, and the protocols and questionnaires will be kept in a locked and secure cabinet particular for this project. Data will be entered in UHeads, a secure research server that holds optimal standards for storage of clinical data. The data from the 6-d dietary record and weight form, filled out by participants, will be entered into SPSS by non-study investigators and quality checked by double entry of 10%. In case of a mismatch, the paper forms will be used to correct the data entry error. The data will not be publicly available to ensure participant confidentiality in accordance with the ethical clearance. After data entry and coding, a cleaned dataset of all combined data will be stored in an SPSS database on a secure server with access limited to the principal investigators. Prior to any data analysis, the Mommy’s Food project group must approve an application of data utilization and intended authorship. To ensure confidentiality, data will be blinded of any identifying participant information if dispersed to non-project team members. Author eligibility guidelines will adhere to the Norwegian national standards for research ethics [27].

Biological samples collected and information derived from this material will be stored in a specific research biobank at IMR. Participants in the study also consent to the inclusion of the biological material and analytical results in the biobank. Last author (MK) is responsible for the research biobank. The biobank is scheduled to expire in 2025. After that, data material will be anonymized. The biological material may only be used after approval by the Regional Committee for Medical and Health Research Ethics (REK).

### Statistical considerations

#### Sample size calculation

A sample size of 60 women/group will have a 95% power to detect a 30% higher UIC in the intervention group compared to the control group (i.e., median UIC of ~120 μg/L compared with 80 μg/L. A total sample size of 144, divided into two groups, is anticipated, taken into account a 20% drop out rate. Data from the “Little in Norway” cohort has been applied in the power calculation of sample size. The following simplified equation has been used to calculate daily iodine intake and UIC [28]: *UIC / 0.92 x (0.0009 L/h/kg × 24 h/d) x weight (kg) = daily iodine intake.* The median UIC was 82 μg/L and the estimated iodine intake was 114 μg/day. The intervention group in this study will be given 400 g of cod per week, with an approximate iodine content of 100 μg/100 g. This intake will increase the mean estimated intake of iodine per week from 800 μg to 1200 μg.

#### Data analysis

The continuous variables will be summarized as mean ± standard deviation (SD) and the categorical variables will be described in frequency and percent. The baseline characteristics will be compared by either the Student t-test for the continuous variables or χ^2^-test for the categorical data. All analyses will initially be done on an intention-to-treat basis (ITT) and all participants will be included in the analyses if the relevant outcome variables have been collected. A per-protocol analysis will also be conducted for the primary and secondary outcome if there are a considerable number of protocol violators. For the primary outcome, a one-way ANCOVA will be used comparing differences in post-intervention UIC between the intervention group and the control group with the pre-intervention UIC as a covariate, as it is believed that the post-intervention UIC will depend, to some degree, on the pre-intervention UIC. For the secondary outcome, we will use linear regression to compare the mean Bayley-III cognitive score, the language scores (receptive and expressive separately and as a composite score) and the motor scores (fine and gross motor separately and as a composite score) between the two groups. The Bayley-III scores will be used on a continuous scale and is expected to be normally distributed. We will also investigate potential effect modification by other nutrients and confounding factors. In the case of unanticipated differences occurring between the intervention and control group, we will adjust for that/those factor(s) in the analyses for both the primary and secondary outcome. For the other outcomes, we will use a variety of statistical approaches where the data may be used as predictors, mediators or moderators. These analyses will be based on plans of analyses for the specific research questions that are addressed. Multilevel linear modeling will be applied to assess longitudinally data. Statistically significance will be set at *p* < 0.05.

#### Ethics and dissemination

The trial complies with the Declaration of Helsinki, is approved by the Regional Committees for Medical and Health Research Ethics West (2015/879), and is registered in ClinicalTrials.gov (NCT NCT02610959). Infants must be especially protected in research since they do not have the competence to give informed consent and ensure that their interests are taken proper care of. Written informed consent was obtained from participants by study investigators after both written and oral information of the study. Participants can withdraw from the study at any time without giving any reason. If a participant wishes to withdraw from the study, they may require to delete collected samples and information unless the information is already included in analyzes or used in scientific publications.

It is optional to provide biological samples. All tests are performed in an age adequate way for the infants. The only invasive procedure is sampling of venous blood. Analysis have been carefully chosen regarding sample material to minimize volume drawn from the infants. Blood sampling will be performed by trained phlebotomist with experience in blood sampling of infants and children.

The mother will be informed if she or her infants’ thyroid hormone status, iron status or vitamin B_12_ status is outside the normal range and be referred to her family doctor. These parameters will be analyzed in a NS-EN ISO 15189 accredited medical laboratory and thus fulfilled the ethical considerations for notification concerning pathological blood test results. Other parameters will be analyzed at IMR in NS-EN ISO/IEC 17025 laboratories and will thus not fulfill the ethical considerations for notification concerning pathological blood test results. In addition, the samples may be analyzed a considerable time after sampling.

Results from the project will be published in peer-review journals and presented at international and national conferences. During the project period, preliminary results will be presented on conferences and seminars, both orally and as posters. Progress reports and statements will be prepared during the study and after completion, a final report for FHF partners will be prepared. A summary popular scientific article will be published in national magazines or newspapers.

## Discussion

Insufficient iodine status in pregnancy is a public health concern. The Norwegian Health Authorities recently published a report acknowledging the need for urgent action to secure iodine nutrition in the population [29]. The present trial (Mummy’s Food) is, to our knowledge, the first RCT intervening pregnant women with iodine rich fish. Findings from this study are expected to provide knowledge on whether an increased intake of cod during pregnancy will improve maternal iodine status, and if this will have an effect on early child development. Previous observational studies have found associations between prenatal iodine nutrition and cognitive functioning in general, as well as on specific developmental areas such as language [8, 30]. The present study will be able to confirm these associations between iodine and development, and test whether the same areas of development are impacted by the dietary intervention. This RCT will also provide valuable research data on other hypotheses regarding methodological considerations, maternal and infant nutrition, mental health and infant development. In case of future funding, the participants will be asked to complete a developmental testing at about five years of age.

### Trial status

Enrolment has closed with 137 participants. The intervention ended in September 2017. Completion of follow up at six weeks, three months-, six months, and eleven months are December 2017, January 2018, April 2018, and September 2018, respectively. This is protocol version number 01, issue date 17 January 2018.

## Abbreviations

ASQ:SE: The ages and stages questionnaire: social emotional
ASQ-3: The Ages and Stages questionnaire, 3rd edition
Bayley-III: The bayley scale of infant and toddler development, 3rd edition
BISQ: The brief infant sleep questionnaire
EFSA: The European Food Safety Authority
EPDS: The Edinburgh postnatal depression scale
FD: Food diary
FFQ: Food frequency questionnaire
HADS: The hospital anxiety and depression scale
Hg: Mercury
ICP-MS: Inductively coupled plasma mass spectrometry
IMR: Institute of marine Research
ITT: Intention to treat
JECFA: The Joint FAO/WHO expert committee on food additives
MoBa: The Norwegian mother and child cohort study
PCBs: Polychlorinated biphenyls
RU: Routine ultrasound
SNP: Single nucleotide polymorphisms
T3: Triiodothyronine
T4: Thyroxine
TEQ: Toxic equivalences
UFGC: Ultrafast gas chromatographic
UIC: Urinary iodine concentration
